# Immune Response Is Key to Genetic Mechanisms of SARS-CoV-2 Infection With Psychiatric Disorders Based on Differential Gene Expression Pattern Analysis

**DOI:** 10.3389/fimmu.2022.798538

**Published:** 2022-02-04

**Authors:** Jing Xia, Shuhan Chen, Yaping Li, Hua Li, Minghong Gan, Jiashuo Wu, Clare Colette Prohaska, Yang Bai, Lu Gao, Li Gu, Dongfang Zhang

**Affiliations:** ^1^ Department of Pharmacognosy, School of Pharmacy, China Medical University, Shenyang, China; ^2^ Institute of Medicinal Plant Development, Chinese Academy of Medical Sciences & Peking Union Medical College, Beijing, China; ^3^ Division of Pulmonary, Critical Care, Sleep, and Occupational Medicine, Department of Medicine, Indiana University, Indianapolis, IN, United States; ^4^ Department of Clinical Pharmacology, School of Pharmacy, China Medical University, Shenyang, China

**Keywords:** SARS-CoV-2, psychiatric illness, differentially expressed gene, COVID-19, functional enrichment

## Abstract

Existing evidence demonstrates that coronavirus disease 2019 (COVID-19) leads to psychiatric illness, despite its main clinical manifestations affecting the respiratory system. People with mental disorders are more susceptible to COVID-19 than individuals without coexisting mental health disorders, with significantly higher rates of severe illness and mortality in this population. The incidence of new psychiatric diagnoses after infection with severe acute respiratory syndrome coronavirus 2 (SARS-CoV-2) is also remarkably high. SARS-CoV-2 has been reported to use angiotensin-converting enzyme-2 (ACE2) as a receptor for infecting susceptible cells and is expressed in various tissues, including brain tissue. Thus, there is an urgent need to investigate the mechanism linking psychiatric disorders to COVID-19. Using a data set of peripheral blood cells from patients with COVID-19, we compared this to data sets of whole blood collected from patients with psychiatric disorders and used bioinformatics and systems biology approaches to identify genetic links. We found a large number of overlapping immune-related genes between patients infected with SARS-CoV-2 and differentially expressed genes of bipolar disorder (BD), schizophrenia (SZ), and late-onset major depressive disorder (LOD). Many pathways closely related to inflammatory responses, such as MAPK, PPAR, and TGF-β signaling pathways, were observed by enrichment analysis of common differentially expressed genes (DEGs). We also performed a comprehensive analysis of protein–protein interaction network and gene regulation networks. Chemical–protein interaction networks and drug prediction were used to screen potential pharmacologic therapies. We hope that by elucidating the relationship between the pathogenetic processes and genetic mechanisms of infection with SARS-CoV-2 with psychiatric disorders, it will lead to innovative strategies for future research and treatment of psychiatric disorders linked to COVID-19.

**Graphical Abstract:**
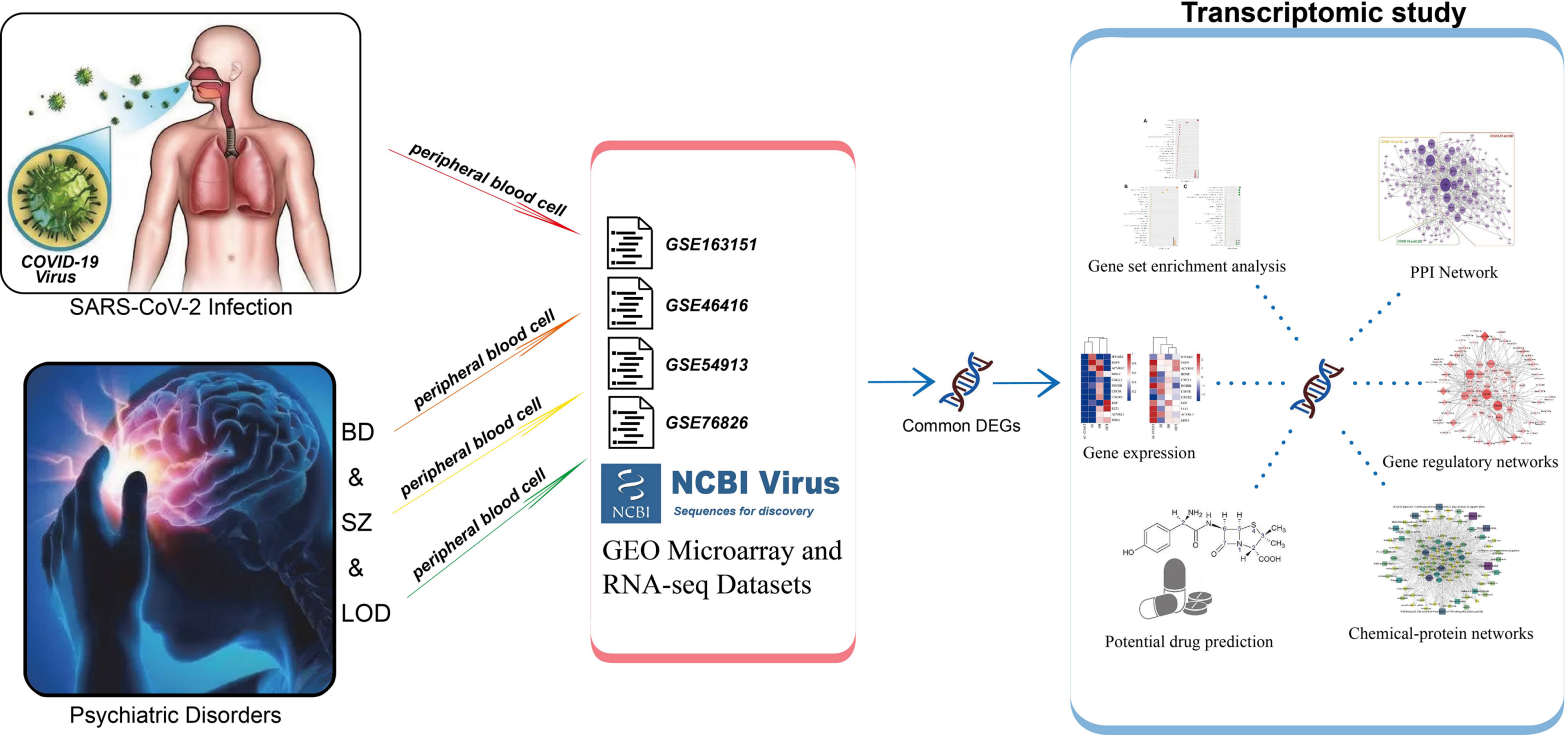


## Introduction

The novel coronavirus disease 2019 (COVID-19) caused by the highly transmissible severe acute respiratory syndrome coronavirus 2 (SARS-CoV-2) is the biggest health problem facing the world today (1). Its severe infectivity and fatality rate prompted the World Health Organization (WHO) to declare it a pandemic in March 2020. COVID-19 is a threat to human health and is wreaking havoc on other areas of daily life, such as social interactions and the global economy (2, 3). Globally, by August 2021, there were about 210 million confirmed cases of COVID-19, including 4.4 million deaths (4). There is some evidence that the determining factors of the severity of infection with SARS-CoV-2 are more possibly related to host factors but not viral genetic variation (5), which has been confirmed by several clinical studies. Pre-existing conditions, such as lung disease, cardiovascular disease, kidney disease, type 2 diabetes, hypertension, and psychiatric illness, significantly increase the likelihood of severe symptoms as well as mortality in patients with COVID-19 (5–10). In a recent study, the prevalence of schizophrenia was 3.6% in 7,341 patients with COVID-19, much higher than the 0.66% prevalence in the general population (11–13). Previous studies have shown that people with mental illness are two to seven times more likely to die after a respiratory infection, and severe mental health disorders have been shown to be a risk factor for increased mortality from COVID-19 (13–15). Furthermore, extensive studies indicate that while COVID-19 is damaging to the respiratory system, it can also have negative mental health effects. Patients with COVID-19 initially show symptoms of headache, fatigue, unsteady gait, with severity depending on age, obesity, mental status, and other health-related conditions (16). COVID-19 may be further complicated by neurological conditions, including polyneuritis, Guillain–Barre syndrome, and encephalomyelitis (17). Due to the fact that preexisting psychiatric disorders elevate the risk of severe illness and death in COVID-19, it is necessary to explore how these disorders are related to COVID-19 infection. Hopefully, future treatments for SARS-CoV-2 infection can ameliorate these complications by modulating pathways between the disease and COVID-19.

COVID-19 is caused by SARS-CoV-2, which belongs to the coronavirus family (15, 18). Its pathogenicity is effected *via* the angiotensin-converting enzyme-2 receptor (ACE2) (19–21). Studies have shown that ACE2 greatly promotes the efficiency of SARS-CoV-2 replication. The ability of SARS-CoV-2 to gain intracellular entry depends on the presence of ACE2 protein (22), leading to active infection (23). The ACE2 receptor is present in the lungs, cardiovascular system, gut, kidney, liver, brain, and adipose tissue (24, 25), as well as the medulla oblongata, glial cells, and central nervous system (17, 19, 26). ACE2 has a variety of functions in the central nervous system, with significant regulatory roles in stress response, anxiety, cognition, brain damage, and neurogenesis (27, 28). Thus, ACE2 may be the key to potentially shared mechanisms underlying COVID-19 and psychiatric disorders.

Herein, we explored the cross talk between COVID-19 and bipolar disorder (BD), schizophrenia (SZ), and late-onset major depressive disorder (LOD). BD is characterized by alternating hypomanic and manic episodes and is among the most serious mental health disorders globally, typically associated with an early age of onset (29, 30). SZ is a multifactorial neuropsychiatric disorder attributed to a developmental disorder of the brain, with symptoms usually occurring during adolescence through young adulthood (31, 32). LOD refers to major depressive disorder that manifests typically after the age of 50 with depressive symptoms and characteristics different from those of early-onset patients (33, 34). We used data sets for COVID-19 and each psychiatric disorder to obtain common differentially expressed genes (DEGs) and common immune DEGs. Through the analysis of these DEGs, we explored the pathway and gene expression ontologies to study their biological significance. Protein–protein, transcription, and post-transcription expression interaction networks were built with common DEGs. Chemical–protein interaction and drug prediction for comorbidities were screened.

## Materials and Methods

### Workflow Overview

Bioinformatics along with system biology methods is commonly used to study comorbidity complexities (35–37). We collected data sets from a public data resource. To determine the DEGs in each data set, gene expression analysis was performed. Common DEGs between COVID-19 and BD, SZ, and LOD were identified. These common DEGs were then used to establish protein–protein interaction (PPI) networks and identify cell signaling pathways and functional gene ontology (GO) to investigate the biological significance. We used common DEGs to create gene regulatory networks (GRN): DEG–microRNA (miRNA) network and DEG–transcription factor (TF) network. Lastly, we identified protein–chemical interactions and screened suggested drugs. The overall workflow is depicted in **Table 1**.

**Table 1:** A summary of the conducted experiments to determine comorbidity complexities shared among psychiatric disorders and COVID-19.

Experiments	Input	Materials/methods	Presented in	Findings
Identification of DEG	Data sets of disorders with a list of genes	Condition used: adjusted *P*-value <0.05 and absolute value of log_2_ fold-change ≥1	Volcano plots, Venn diagrams, heatmaps	List of DEGs
PPI	Shared DEGs between COVID-19 and psychiatric disorders	STRING (38)	PPI interaction network	Interaction among proteins
Pathway analysis	Shared DEGs between COVID-19 and each of the psychiatric disorders	KOBAS-i (39)	Bubble plots	List of significant pathways
GO	Shared DEGs between COVID-19 and each of the psychiatric disorders	KOBAS-i (39)	Bubble plots	List of significant GO pathways
DEG–miRNA interaction	Shared DEGs between COVID-19 and each of the psychiatric disorders	Network Analyst (40)	Networks of DEGs–miRNAs	Interactions between DEGs and miRNAs
DEG–TF interaction	Shared DEGs between COVID-19 and each of the psychiatric disorders	Network Analyst (40)	Networks of DEGs–TFs	Interactions between DEGs and TFs
Drug prediction	Shared DEGs between COVID-19 and each of the psychiatric disorders	L1000FDW (41)	Potential drugs table	List of drugs
Protein–chemical interaction	Shared DEGs between COVID-19 and each of the psychiatric disorders	Network Analyst (40)	Networks of proteins–chemicals	Interactions between protein–chemical agents

### Gene Expression Data Set

We collected selected human gene expression raw data sets for BD, SZ, and LOD from the Gene Expression Omnibus (GEO) of the National Center for Biotechnology Information (NCBI). The GEO accession number of the COVID-19 data set is GSE163151, and the samples of this data set were obtained from whole peripheral blood cell samples of individuals with COVID-19 along with healthy control subjects. Samples of selected psychiatric disorders data sets having association numbers GSE46416 (BD patients and healthy controls), GSE54913 (SZ patients and healthy controls), and GSE76826 (LOD patients and healthy controls) were collected by analyzing the peripheral blood of the patients and healthy controls.

### Identification of DEGs

For the purpose of determining the DEGs in data sets, we used the limma R package, to identify DEGs (42). The Benjamini–Hochberg false discovery rate method can effectively discover genes which are statistically significant and limit false positives. Genes exhibiting an adjusted *P* of <0.05 along with a log_2_ fold-change of ≥1 are determined as DEGs. DEGs of all data sets were identified in this study, including one for SARS-CoV-2 and three for psychiatric disorders. The shared DEGs in psychiatric illnesses and SARS-CoV-2 were also screened. Additionally, DEGs which are shared in SARS-CoV-2 immune and psychiatric illness were determined by ImmPort (http://www.immport.org/).

### Gene Set Enrichment Analysis

Gene set enrichment analysis, which can clarify potential biological mechanisms, consisted of pathway analysis and gene ontology analysis (9, 39). Pathways with the adjusted *P*-value <0.05 were eligible. We selected pathway and ontology terms by KOBAS-i (http://kobas.cbi.pku.edu.cn/) (39). Pathways were obtained from four data resources including Reactome, KEGG, Panther, and BioCyc in KOBAS-i.

### Protein–Protein Interaction Analysis

The PPI (38) is commonly used to explain the association between diseases, so we utilized DEGs to create PPI networks. We used version 11.5 of STRING (https://www.string-db.org/) to insert common DEGs to generate PPI networks. The confidence score was used for the PPI network using the STRING platform with a medium confidence score of 0.400. We performed the Markov cluster algorithm (MCL) function in STRING to identify gene clusters. For a superior visual representation of the network and for the purpose of identifying hub genes, the PPIs are analyzed through Cytoscape (https://cytoscape.org/).

### Gene Regulatory Network Analysis

We used GRN to explore the underlying mechanisms of disease. DEG–miRNA cross talk networks and DEG–TF cross talk networks from common DEGs were constructed by Network Analyst (40). TarBase (43) and miRTarBase (44) data resources were employed for DEG–miRNA cross talk networks, and the JASPAR (45) data resource was used in TF–DEG cross talk network analysis. DEG–miRNA and DEG–TF networks were filtered with a betweenness value of 150 and 4,000, respectively, and were illustrated in Cytoscape. Regulation at the post-transcriptional level was identified by these interaction networks.

### Chemical Compound and Suggested Drug Analysis

We used common DEGs between COVID-19 and psychiatric illnesses to construct the chemical–protein interaction networks and a list of suggested therapeutic drugs. The chemical–protein interaction networks from common DEGs were built by the Comparative Toxicogenomics Database (CTD) in Network Analyst (40). The networks were filtered with a degree value of 10 and were illustrated in Cytoscape. The list of drugs derived by common DEGs was identified by the web-utility L1000FDW (https://maayanlab.cloud/L1000FWD/) (41), which can determine potential repurposable pharmacological agents. An adjusted *P*-value (*q*-value) <0.05 was used as a threshold for significance. Among them, the top 5 drugs which have been marketed are shown in our results.

### Statistical Analysis

DEGs were identified for each data set by using adjusted *P*-values (adj *P*) <0.05 along with an absolute value of logFC (log fold-change) of >1.

## Results

### Differentially Expressed Genes in Blood Demonstrate Genetic Associations Between COVID-19, BD, SZ, and LOD

**Figure 1:**
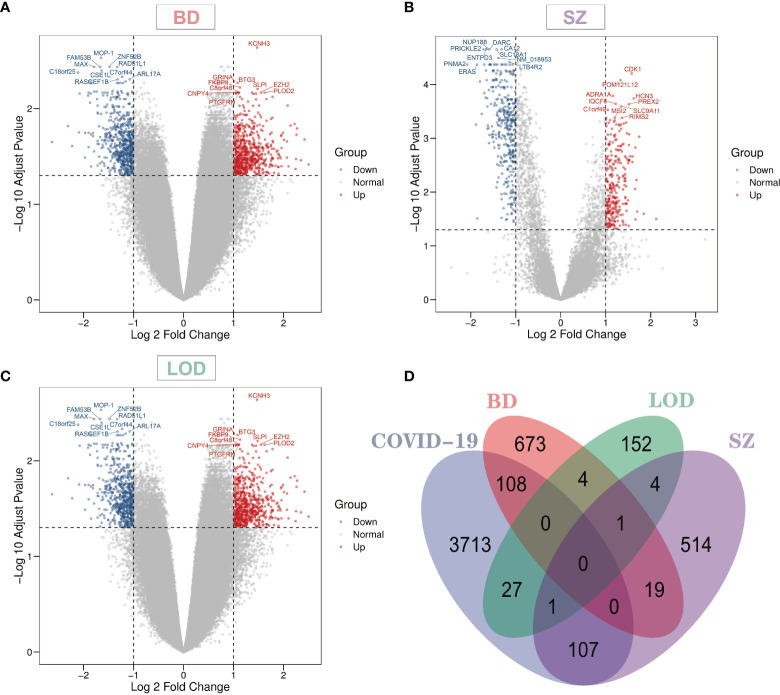
Volcano plots indicate differentially expressed genes (DEGs) of **(A)** bipolar disorder (BD), **(B)** schizophrenia (SZ), and **(C)** late-onset major depressive disorder (LOD), with genes harboring log fold-change of at least 1 and adjusted *P*-value <0.05. The Venn diagram depicts the shared DEGs among coronavirus disease 2019 (COVID-19) and **(D)** BD, SZ, and LOD.

Overall, 3,956 DEGs were identified from COVID-19 blood data sets. The identified DEG numbers for BD, SZ, and LOD were 805, 646, and 189, respectively (**Supplementary Table 1**). **Figure 1** demonstrates notable genes for SZ, BD, and LOD. The red dots in the volcano plots designate genes remarkably upregulated and the blue dots designate remarkably downregulated genes. The top 10 gene names with the lowest adj *P*-value are represented expressly in the figure. We conducted a comparative assessment to determine DEGs that are shared in COVID-19 with other diseases. **Figure 1D** illustrates the number DEGs shared in these conditions. Our results reveal that shared DEGs with COVID-19 are more common in SZ and BD but not with LOD. Of particular note is *CELSR1*, a common differential gene of COVID-19, SZ, and LOD.

### Common DEGs for Immune Responses of COVID-19, BD, SZ, and LOD

**Figure 2:**
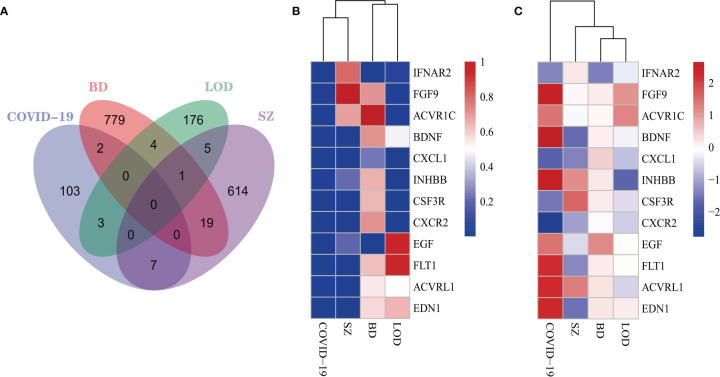
**(A)** The Venn diagram illustrates the common DEGs in COVID-19 immune system and BD, SZ, and LOD. Heatmaps demonstrate the associations of DEGs based on **(B)** adjusted *P*-value and **(C)** log fold-change for all immune data sets.

We identified DEGs from immune response gene sets from patients with COVID-19, BD, SZ, and LOD and identified DEGs common among them (**Figure 2A**), with heatmaps demonstrating the relationship between these common DEGs. To reflect the relationship between unaffected and disease states, we constructed heatmaps from two different perspectives: adj *P*-value (**Figure 2B**) and the values of log fold-change (**Figure 2C**). The COVID-19 immune response data set shares more DEGs with SZ in contrast to BD or LOD. The DEGs shared between the immune responses of COVID-19 and SZ consist of *ACVRL1*, *BDNF*, *CSF3R*, *CXCL1*, *CXCR2*, *EDN1*, and *FLT1*. DEGs with COVID-19 and LOD include *ACVR1C*, *FGF9*, and *INHBB*. COVID-19 immune response and BD share two DEGs, *EGF* and *IFNAR2*.

### Functional Enrichment Assessment Uncovers Important Cascades and GO Terms

**Figure 3:**
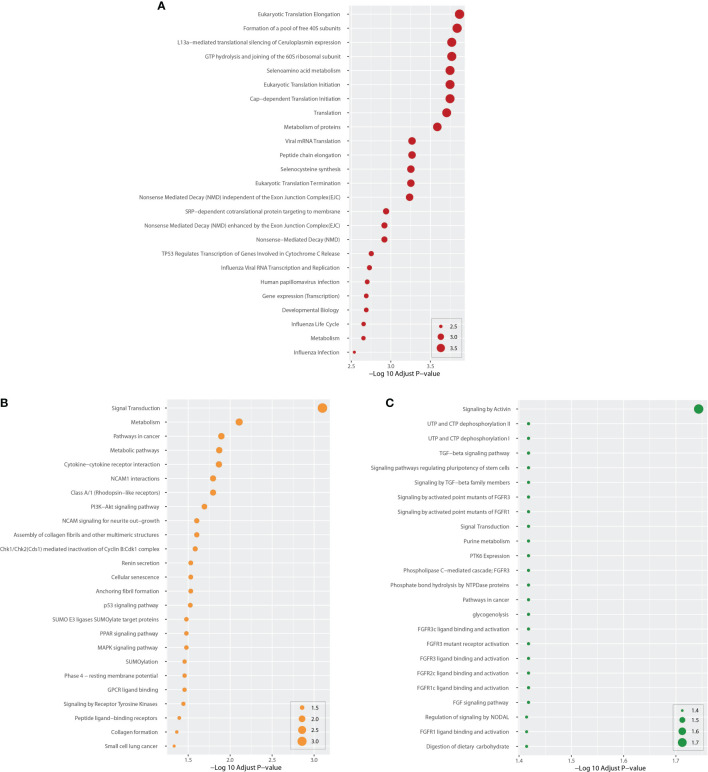
Top 25 cascades between COVID-19 and psychiatric diseases. Cascades were determined using DEGs for each condition and COVID-19 whole blood and immune samples. Panel **(A)** shows cascades for BD. Panels **(B, C)** show pathways for SZ and LOD, respectively. The higher the log adj *P*, the more significant the enrichment.

We identified DEG-related pathways shared by COVID-19 and BD, SZ, and LOD, analyzing the possible roles of these genes in the involved pathways using enrichment analysis combining all DEGs from peripheral blood from patients with COVID-19. After integrating the pathways in these data resources, we plotted the bubble map of the top 25 most significant pathways according to adjusted *P*, as illustrated in **Figure 3**. For example, Pathways Eukaryotic Translation Elongation, Signal Transduction, and Signaling by Activin are the most significantly enriched pathways in BD, SZ, and LOD, respectively (**Figure 3**). As exhibited in **Figure 3A** and **Supplementary Table 2**, there are many pathways associated with the pathogenic mechanisms of various viruses, i.e., viral mRNA translation, influenza viral RNA transcription along with replication, human papillomavirus infection, influenza life cycle, and influenza infection in COVID and BD. In addition, many pathways closely related to inflammatory responses were observed. For instance, the PI3K–Akt signaling cascade, PPAR signaling cascade, and MAPK signaling cascade were found to be closely related in both COVID and SZ (**Figure 3B** and **Supplementary Table 3**). We also found many pathways related to TGF-β and FGF/FGFR signaling between COVID and LOD (**Figure 3C** and **Supplementary Table 4**).

**Figure 4:**
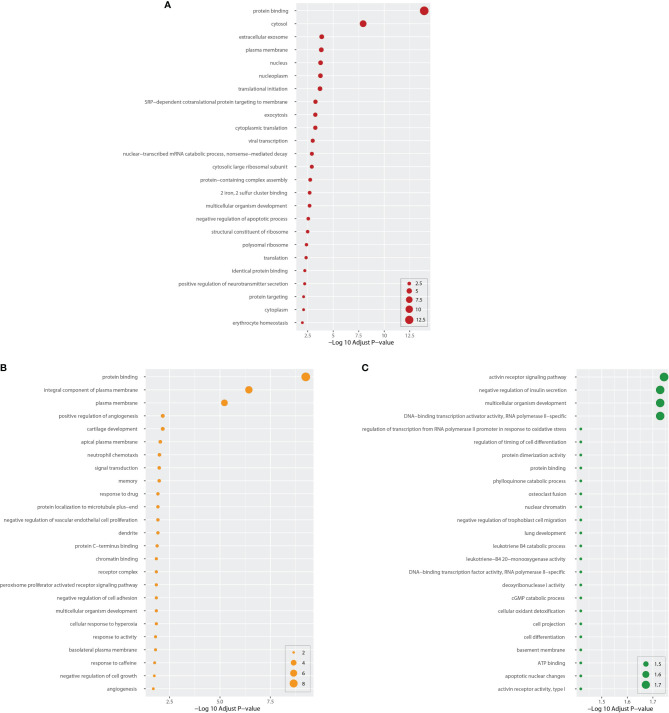
Top 25 gene ontology between COVID-19 and mental disorders. The ontology cascades were determined using the DEGs for each of the condition and the combined genes of COVID-19 whole blood and immune samples. Panel **(A)** exhibits the ontology for BD. Panels **(B, C)** exhibit ontology cascades for SZ and LOD, respectively.

Next, we performed GO pathway analysis using the same common DEGs to explore biological process categories. **Figure 4** shows the top 25 GO pathways of COVID-19 relative to BD, SZ, and LOD. The significantly enriched GO terms are identified by high logarithmic value of the adj *P*-value. Based on **Figure 4**, protein binding is a prominent ontology pathway between COVID-19 and BD. This GO pathway was also significantly enriched in SZ and LOD (**Supplementary Tables 5**–**7**).

### Protein–Protein Interaction Analysis Identifies Functional Networks

**Figure 5:**
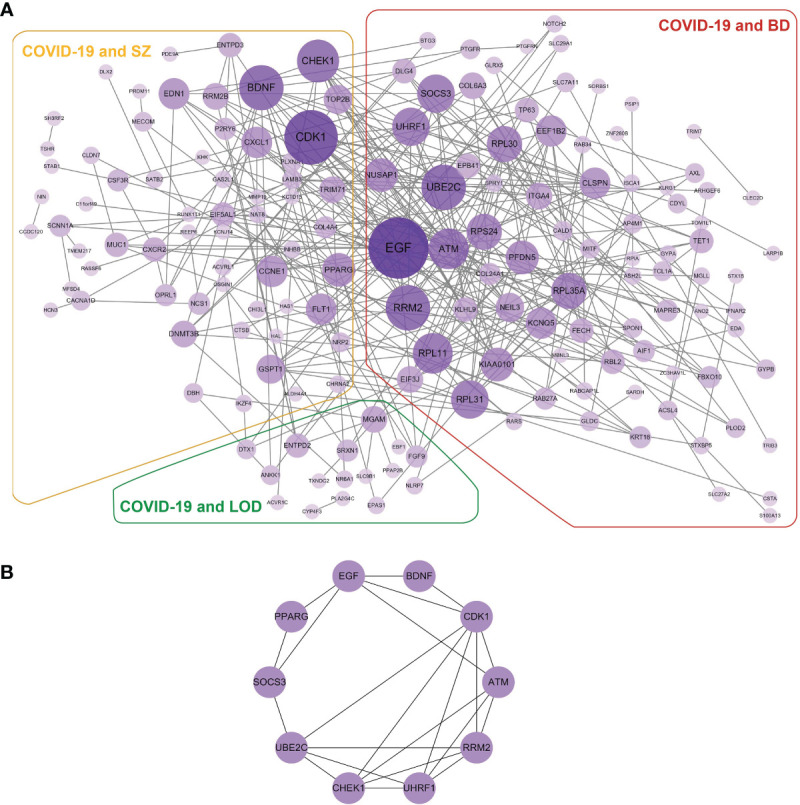
**(A)** The detailed PPI network of shared DEGs in COVID-19 and psychiatric disorders. The purple circles illustrate the proteins common in COVID-19 and corresponding diseases. Nodes designate proteins and edges exhibit cross talk between two proteins. Proteins having multiple edges are overexpressed. **(B)** Hub protein network exhibits eight hub proteins based on the degree of cross talk. EGF has the highest cross talk with other proteins.

We built a PPI network with the shared DEGs between COVID-19 and BD, SZ, and LOD. As illustrated in **Figure 5A**, the PPI network is created by shared DEGs in whole blood along with immune response cells from individuals infected with COVID-19, as well as patients with BD, SZ, and LOD. The purple circles indicate proteins engaged in each of the psychiatric disorders, as well as COVID-19. The cross talk between proteins is designated by the edges. The size and color depth of the circles designate the degree of protein interrelatedness (number of connecting edges), with stronger relationships indicated by more connections with hub proteins (46). **Figure 5B** is a network of hub proteins and their interaction among each other. It is generated using cytoHubba (47) package of Cytoscape. CDK1 has the highest connectivity in the PPI network. UBE2C, EGF, ATM, RRM2, UHRF1, and CHEK1 are also overexpressed among these proteins.

### Gene Modulatory Network Analysis Identifies DEG–miRNA and DEG–TF Interactions

**Figure 6:**
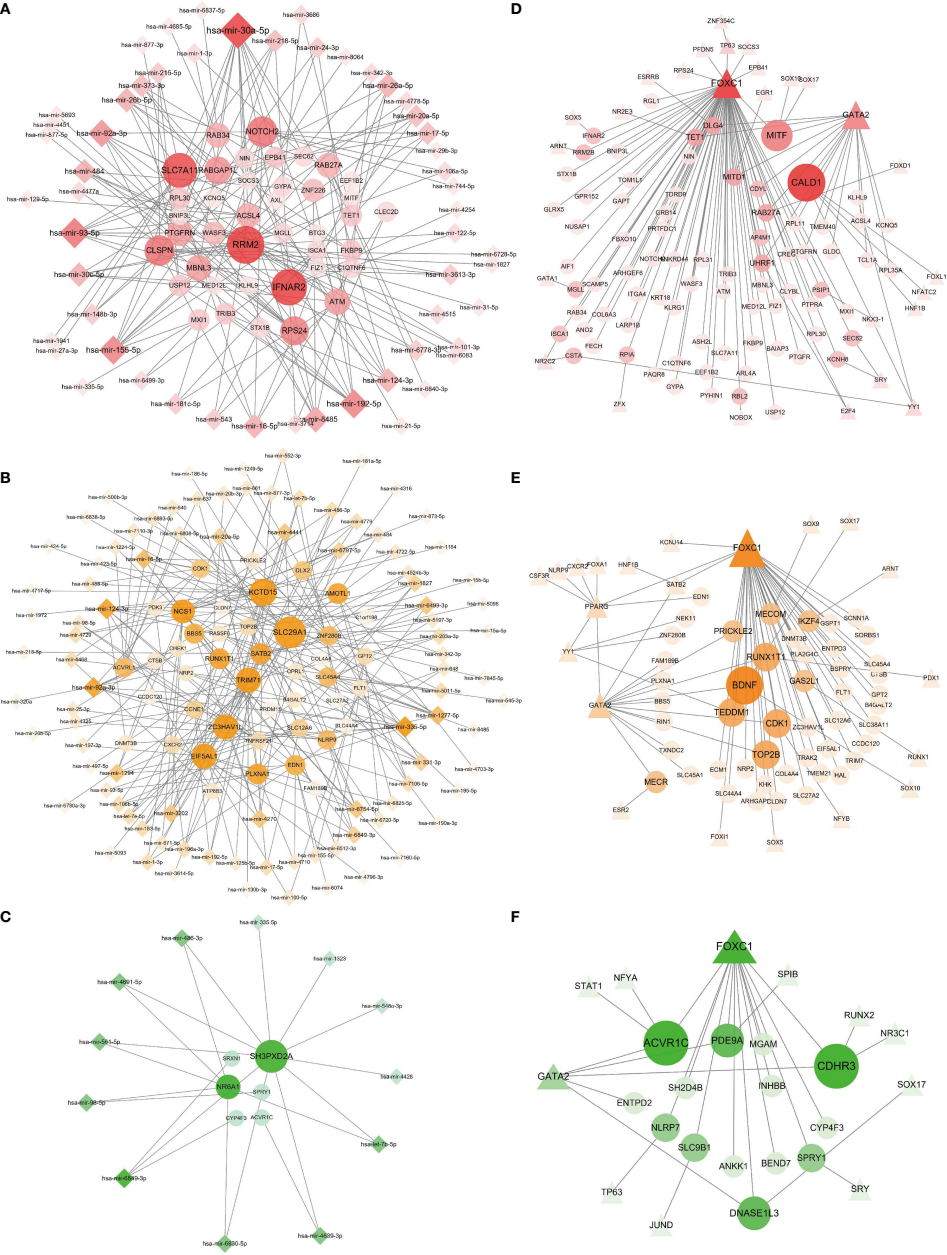
Regulatory gene networks of COVID-19 with psychiatric disorders. Panels **(A–C)** designate the DEG–miRNA networks of COVID-19 with BD, SZ, and LOD, respectively. Circles designate DEGs and diamonds designate miRNAs. The results of the DEG–TF networks of **(D)** BD–COVID-19, **(E)** SZ–COVID-19, and **(F)** LOD–COVID-19. Circles illustrate DEGs, and triangles represent TFs. Overexpressed DEGs, miRNAs, and TFs are designated by larger size and darker color.

We obtained a clear network of interactions between DEG and miRNA by using TarBase and miRTarBase data resources, as shown in **Figure 6**. Circles in the figure designate DEGs, and diamonds indicate miRNAs. One circle is connected with more than one diamond, with the node degree referring to connection numbers between the node and other nodes in the network. Circles and diamonds in **Figure 6** are sized and colored to indicate the degree of the nodes, with the larger the size and the darker the color of a node, the more nodes connected to it, and such nodes are considered to have a critical role in the network. We found that in the BD–COVID-19 interaction (**Figure 6A**), genes *RRM2*, *IFNAR2*, and *SLC7A11* and miRNAs hsa-mir-30a-5p, hsa-mir-93-5p, and hsa-mir-192-5p have more significant effects.

As displayed in **Figures 6D–F**, we also constructed an interaction network between DEGs and transcription factors (TF) through the JASPER data resource, using the same DEGs as the DEG–miRNA network. Circles and triangles designate DEGs and TFs, respectively. Likewise, the size of the circles and triangles, as well as their shades of colors, depends on the degree of the nodes. **Figure 6E** illustrates the DEG–TF network of BD–COVID-19 demonstrating that *BDNF*, *RUNX1T1*, *CDK1*, *TOP2B*, and *TEDDM1* constitute the overexpressed genes, and FOXC1 and GATA2 are among the significant TFs.

### Chemical Compounds and Drug Prediction Analysis Identifies Chemical–Protein and Protein–Drug Interactions

**Figure 7:**
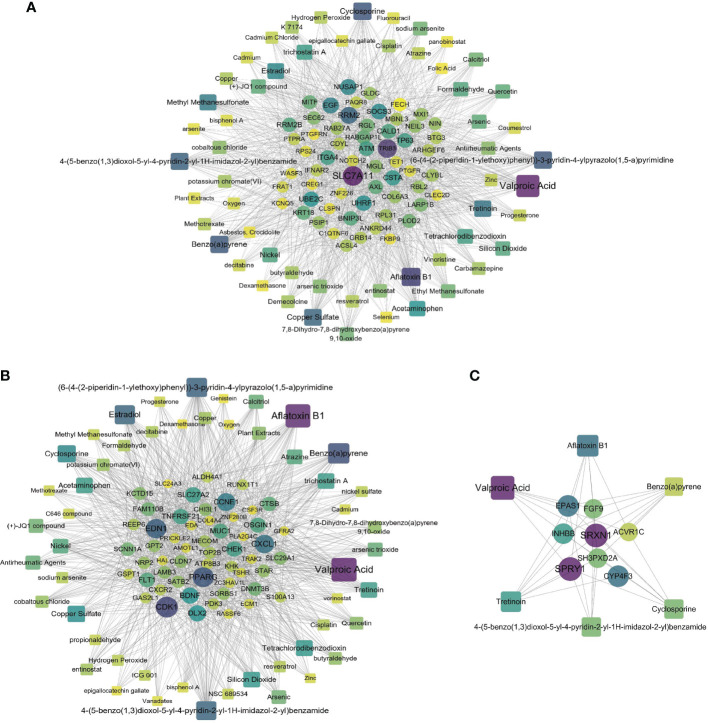
Relationship of COVID-19 and psychiatric disorders with regard to chemical and protein agents. Panels **(A–C)** illustrate the chemical–protein networks of COVID-19 with BD, SZ, and LOD, respectively. Circles designate proteins and squares designate chemical compounds. Overexpressed proteins and chemicals are designated by larger size and darker color. Squares having multiple edges are the most overexpressed chemical agents.

A chemical–protein interaction network is an important research tool for understanding the function of proteins, which is helpful for advancing drug discovery. We created a chemical–protein cross talk network using shared DEGs for COVID-19 with BD, SZ, and LOD using peripheral blood and immune cells, shown in **Figure 7**. The squares in **Figure 7** designate chemicals that influence the expression of the gene, and round nodes represent protein-coding genes. The degree of the node reflects the number of connections the node harbors with other nodes in the network. The color from yellow to purple and node size indicate the degree of node from low to high. **Figure 7A** illustrates the chemical–protein cross talk network between BD and COVID-19. Significant proteins in this network include SLC7A11, TRIB3, RRM2, EGF, and NUSAP1. **Figure 7B** illustrates the chemical–protein network between COVID-19 and SZ. The overexpressed proteins in this cross talk include CDK1, PPARG, EDN1, CXCL1, and CCNE1. **Figure 7C** shows the overexpressed proteins between LOD and COVID-19, including SRXN1, SPRY1, EPAS1, and CYP4F3. Valproic acid is the most highly enriched chemical observed in BD, SZ, and LOD networks with COVID-19. **Table 2** shows the predicted potential drugs that could treat COVID-19 patients with BD, SZ, or LOD, arranged based on similarity scores. The top drug with the most anti-similar signature for BD was capsaicin. The top drug for SZ was racecadotril. Erythromycin was the most significant drug for LOD.

**Table 2:** Top 5 drugs predicted for use in COVID-19 with BD, SZ, or LOD.

	Drug	Similarity score	*P*-value	*q*-value	*Z*-score	Combined score
COVID-19–BD	Capsaicin	0.0964	0.000113	0.155	−1.67	6.61
	Tacrolimus	0.0964	0.000121	0.155	−1.73	6.77
	Gossypol	0.0843	0.001240	0.296	−1.62	4.71
	Raloxifene	0.0843	0.000597	0.261	−1.85	5.95
	Elesclomol	0.0843	0.000544	0.261	−1.68	5.50
COVID-19–SZ	Racecadotril	0.1026	0.000054	0.425	−1.79	7.64
	Xylazine	0.0897	0.000378	0.569	−1.65	5.66
	Fluocinolone	0.0769	0.002740	0.860	−1.64	4.21
	Halcinonide	0.0769	0.002090	0.860	−1.63	4.36
	Halometasone	0.0769	0.002990	0.860	−1.71	4.33
COVID-19–LOD	Erythromycin	0.2667	0.000080	0.036	−1.68	6.90
	Vincristine	0.2667	0.000135	0.036	−1.61	6.24
	Lobendazole	0.2667	0.000096	0.036	−1.80	7.24
	Cefixime	0.2667	0.000122	0.036	−1.62	6.36
	Pemoline	0.2667	0.000102	0.036	−1.81	7.22

## Discussion

The focus of this paper is to explore the pathogenetic processes and genetic mechanisms between COVID-19 and psychiatric disorders from different biological perspectives. Recently, transcriptome data have often been used in studies related to comorbidities of COVID-19 (9, 10). Through a comprehensive analysis of multiple transcriptomic data, we found that the most shared DEGs were found between COVID-19 with BD and SZ, while only 28 shared DEGs were found between COVID-19 and LOD. Only one gene, Cadherin EGF LAG Seven-Pass G-Type Receptor 1 (*CELSR1*), has been found to be shared between COVID-19 and SZ and LOD. *CELSR1* is related to B-cell proliferation, cell–cell adhesion, and central nervous system development (48). Many clinical manifestations are associated with these processes. Immune cells in COVID-19 patients produce inflammatory cytokines such as IL-2 and IL-6 to stimulate B-cell proliferation (49). Infection with SARS-CoV-2 also causes the increased expression of endothelial cell adhesion molecules, inducing dysfunction of the coagulation cascade (50).

We also analyzed immune responses of individuals with COVID-19 with BD, SZ, and LOD to understand how COVID-19 affects the immune system response process in patients with psychiatric disorders. One hundred and fifteen DEGs from our COVID-19 immune response data set determined by ImmPort were identified. COVID-19 immune response and SZ share one DEG (activin A receptor type II-like 1, or *ACVRL1*) that is upregulated and two DEGs (*CXCR2*, *CXCL1*) that are both downregulated, all of which are strongly associated with the pathogenesis of COVID-19. *ACVRL1* belongs to the family of TGF-β I receptors, and its abnormal changes significantly affect the regulation of the TGF-β pathway, contributing to the development of pulmonary hypertension (51, 52). The imbalance of TGF-β biological activity predisposes individuals to infection with COVID-19 (53). C-X-C chemokine receptor type 2 protein (CXCR2) and its ligand C-X-C motif chemokine 1 (CXCL1) are involved in various inflammatory diseases (54–58). In COVID-19 patients, CXCL1 binds to CXCR2 to promote immune cell chemotaxis, causing a cytokine storm that worsens the symptoms of patients (59, 60). One upregulated DEG (*EGF*) and one downregulated DEG (*IFNAR2*) have been uncovered in COVID-19 responses in BD. The Interferon receptor 2 (*IFNAR2*) gene is involved in the synthesis of interferon which has antiviral effects, with low gene expression levels in patients with severe COVID-19 (61).

In order to illustrate the relationship of mechanisms between COVID-19 and BD, SZ, and LOD, we performed enrichment analyses by using DEGs to discover the biological processes of pathways and gene ontology. The common DEGs used for enrichment analysis were derived from the patients with BD, SZ, and LOD and peripheral blood and immune responses from patients infected with COVID-19. Pathways enriched by genes in COVID-19 and BD are found to be related to viral infections, including the influenza virus and human papillomavirus. During the life cycle of the virus, viral RNA transcription, replication, and viral mRNA translation are highly enriched. This is likely to be the main mechanism by which BD patients are susceptible to SARS-CoV-2 (62). Alternatively, we observed that metabolism of proteins and selenoamino acid metabolism are enriched to varying degrees. We identified multiple cell signaling pathways including PI3K–Akt, MAPK, PPAR, and p53 signaling important between COVID-19 and SZ. The PI3K–Akt signaling pathway responds to extracellular signals and promotes metabolism, proliferation, cell survival, growth, and angiogenesis (63–65). The MAPK signaling pathway is also highly enriched, which is related to the regulation of cell growth, differentiation, stress adaptation to the environment, inflammation, and other remarkable cellular physiological/pathological processes (66–69). PPAR signaling modulates the expression of genes that regulate lipid metabolism, fat formation, and maintenance of metabolic homeostasis along with inflammation, and induces anticancer effects in numerous human tumors (70–74). The p53 signaling pathway is also identified, which plays an important role in restoring the proliferation of cells that have DNA damage (75, 76). Moreover, some potential pathways have been noted such as cytokine–cytokine receptor interaction and signaling by receptor tyrosine kinases. TGF-β and fibroblast growth factors (FGF) and fibroblast growth factor receptor (FGFR) signaling pathways are very significant for LOD and COVID-19. Members of the TGF-β family are expressed in tissue-specific forms at different time points and, therefore, play an indispensable role in the development, homeostasis, and repair of most tissues in the body (77–79). All immune cells, entailing B cells, T cells, dendritic cells, and macrophages, secrete TGF-β, which in turn negatively modulates the proliferation, differentiation, and activation of immune cells through other cytokines (80–84). In addition, its signal disturbance has been linked to autoimmune diseases, inflammation, and cancer (85). FGFs bind to FGFRs, activating downstream signaling pathways, which play an important role in biological processes promoting or inhibiting division (86–88). TGF-β and FGF/FGFR signaling pathways influence biological processes through their interactions (89–91).

The gene ontology analysis reveals protein binding, exocytosis, positive regulation of neurotransmitter secretion, and protein targeting-related pathways for BD. Neutrophil chemotaxis and negative regulation of cell adhesion pathways are the key ontological biological processes in COVID-19 and SZ. Neutrophils play an important role in both sterile and infective inflammatory responses. As the first-line cells of inflammatory responses, neutrophil chemotaxis is a significant manifestation of the anti-inflammatory ability of the body (92). Cell adhesion plays a pivotal role in maintaining the stability of bronchial epithelium mediating airway inflammation in acute and chronic lung inflammation (93–96). Increased chemotaxis of inflammatory cells and upregulation of cellular adhesion molecule expression are also present in the response of the body to COVID-19 (97, 98). In addition to lung development, cell differentiation, and regulation of timing of cell differentiation, there are also activin receptor signaling pathway, protein binding, ATP binding, and apoptotic nuclear change ontology pathways common to LOD and COVID-19.

With the purpose of systematically analyzing the interaction between a large number of proteins and understanding the physiological mechanisms of disease states, we created a PPI network. We found that epidermal growth factor (*EGF*), which regulates intracellular signaling molecules, was the highest expressed gene in the interactive network. *EGF* can promote the survival, maturation, and differentiation of neurons, especially midbrain dopaminergic neurons, and its dysfunction may affect the physiological and pathological conditions of BD (99, 100). A previous study on COVID-19 suggested that *EGF* has a potential role in lung injury in infection with SARS-CoV-2 (101). Furthermore, cyclin-dependent kinase 1 (*Cdk1*) has the highest connectivity among hub genes. In response to SARS-CoV-2 infection, the activity of CDK1 and other cycle-related kinases decreased significantly in host cells (102, 103). It plays an indispensable role in regulating mitosis, cell cycle progression, apoptosis, and cell pluripotency (104, 105). The cell cycle-related genes have been used as biomarkers in research investigating the diagnosis and treatment of schizophrenia (106–108). Many overexpressed genes, such as *UBE2C*, *ATM*, *RRM2*, *UHRF1*, and *CHEK1*, are related to immune system function and provide potential mechanisms in SARS-CoV-2 infection (109–113). A further in-depth study of these genes may contribute to the clinical diagnosis and treatment of COVID-19 and its comorbidities with psychiatric disorders.

We have found relationships between DEG–miRNA and DEG–TF interactions in COVID-19 and psychiatric disorders. We identified miRNAs with key roles including hsa-mir-30a-5p, hsa-mir-93-5p, hsa-mir-155-5p, hsa-mir-92a-3p, hsa-mir-335-5p, hsa-mir-124-3p, and hsa-mir-6849-3p. These miRNAs are important in the pathogenesis of mental illnesses and also play a role in other major diseases. For example, hsa-mir-124-3p plays a remarkable role in the development of colon cancer, nasopharyngeal cancer, and gliomas (114). Furthermore, our research indicates that transcription factors FOXC1, GATA2, YY1, NR2C2, and E2F4 were overexpressed in common DEGs of psychiatric disorders and COVID-19. Previous studies have suggested that the functions of the FOX and GATA families affect the pathophysiological mechanisms of psychiatric illnesses (115, 116). Using our chemical–protein interaction networks, we were able to predict drugs by the web-utility L1000FDW to screen candidate drug molecules. Through chemical–protein networks, we found valproic acid, cyclosporine, estradiol, and tretinoin, all of which have potential effects in fighting COVID-19. Valproic acid had shown good clinical value in the treatment of COVID-19 and Alzheimer’s disease (117, 118). Drug prediction results showed that capsaicin, racecadotril, and erythromycin had the strongest effect according to similarity score. In one recent study, capsaicin showed strong activity in treating mental illness (119). In addition, capsaicin and cefixime both have a strong ability to penetrate the blood–brain barrier (BBB) (120, 121), and vincristine can also penetrate the BBB to a certain extent (122, 123). This will provide implications and new ideas for the clinical prevention along with treatment of COVID-19 and its co-occurrence with BD, SZ, and LOD.

## Conclusions

To explore the pathogenetic processes and genetic mechanisms of comorbidities of COVID-19 with psychiatric disorders, we identified common DEGs in BD, SZ, and LOD with COVID-19 and its immune response. Common DEGs in psychiatric disorders with COVID-19 and its immune response have been integrated to identify the pathways and genetic ontological terms. We found that TGF-β, MAPK, PPAR, and other immune-related pathways were highly enriched. We created PPI interaction networks to understand the relationship between important proteins in COVID-19 patients and BD, SZ, and LOD. Hub proteins were identified, with CDK1 being the most highly expressed protein. Transcriptional and post-transcriptional analyses were also performed to uncover the involvement of potential miRNAs and transcription factors and to identify important factors for infection with SARS-CoV-2. Finally, we created chemical–protein networks and drug prediction to identify a number of potentially drug molecules. The genes, pathways, and networks identified in this study are in accordance with previous studies, implying that there are multiple mechanisms of comorbidity between COVID-19 and BD, SZ, and LOD. We hope our work will lead to further investigation and provide new ideas and treatment strategies in the clinical fight against COVID-19.

## Data Availability Statement

The data sets presented in this study can be found in online repositories. The names of the repository/repositories and accession number(s) can be found in the article/**Supplementary Material**.

## Author Contributions

Conceptualization: DZ, LiG, and JX. Supervision: LiG, JX, and SC. Sample collection: YL, HL, JX, and MG. Experiments: YL and JX. Investigation: JX, MG, JW, and HL. Data analysis: JX, YB, and LuG. Visualization: JX and JW. Manuscript draft: JX, SC, and MG. Manuscript revision and editing: CP, YB, LuG, and DZ. All authors contributed to the article and approved the submitted version.

## Funding

This study was financially funded by the National Nature Science Foundation of China (No. 81803530) and the Natural Science Foundation Plan Guidance Project of Liaoning Province (No. 20180550144).

## Conflict of Interest

The authors declare that the research was conducted in the absence of any commercial or financial relationships that could be construed as a potential conflict of interest.

## Publisher’s Note

All claims expressed in this article are solely those of the authors and do not necessarily represent those of their affiliated organizations, or those of the publisher, the editors and the reviewers. Any product that may be evaluated in this article, or claim that may be made by its manufacturer, is not guaranteed or endorsed by the publisher.
